# Haematological alterations in the context of olanzapine treatment

**DOI:** 10.1192/j.eurpsy.2024.1439

**Published:** 2024-08-27

**Authors:** B. Serra, D. Ramis, R. Catalán, V. Llorca

**Affiliations:** Institut Clínic de Neurociències, Hospital Clínic de Barcelona, Barcelona, Spain

## Abstract

**Introduction:**

Haematological alterations, especially in the red blood cell series, are a rare adverse effect of olanzapine treatment. A 64-year-old female patient with a diagnosis of long-standing schizophrenia was admitted to the psychiatric room for psychotic decompensation and leukopenia in control laboratory tests. She had a history of mild psoriasis, allergy to sulphonamides and infectious bursitis nine years earlier secondary to neutropenia due to clozapine. On previous admission, episodes of anaemia and neutropenia related to increased doses of olanzapine were observed. On current admission, a new episode of anaemia and neutropenia occurred with doses of up to 20 mg/day of olanzapine, hemoglobin levels of 63g/L ann neutrophil count of 0,8*10^9 neutrophils/l were detected.

**Objectives:**

Report a very rare but serious adverse effect in patients treated with olanzapine.

**Methods:**

Haematological analysis were periodically carried out from 2009 to 2023.

A complete study was carried out with parameters of haemolysis, autoimmunity, a pharmacogenetic study and a myelogram.

**Results:**

The autoimmunity and haemolysis study excluded an autoimmune or haematological illness that could justify the haematological alterations.

The myelogram showed normal cellularity.

The pharmacogenetic study showed no relevant alterations.

**Image:**

**Figure:**
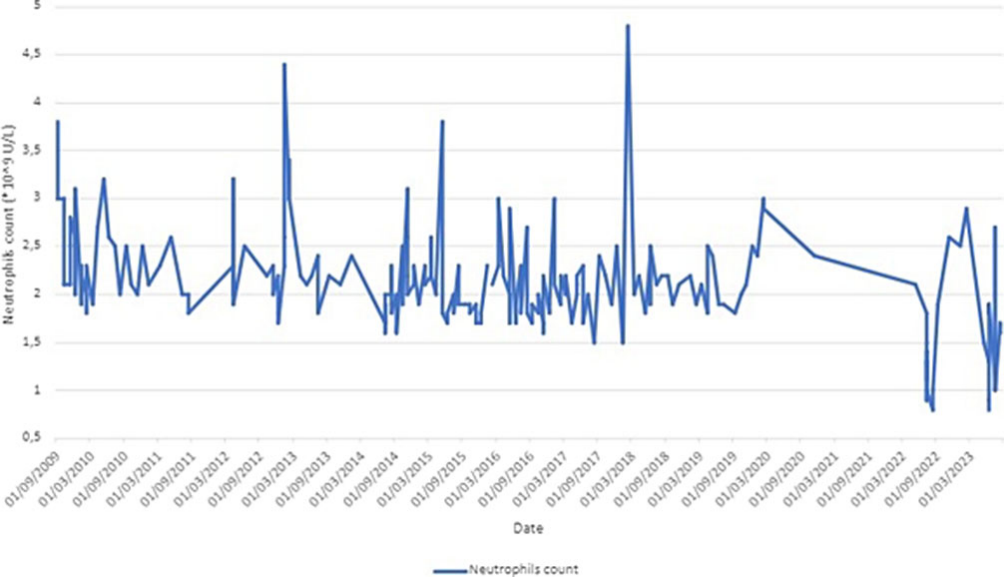

**Image 2:**

**Figure:**
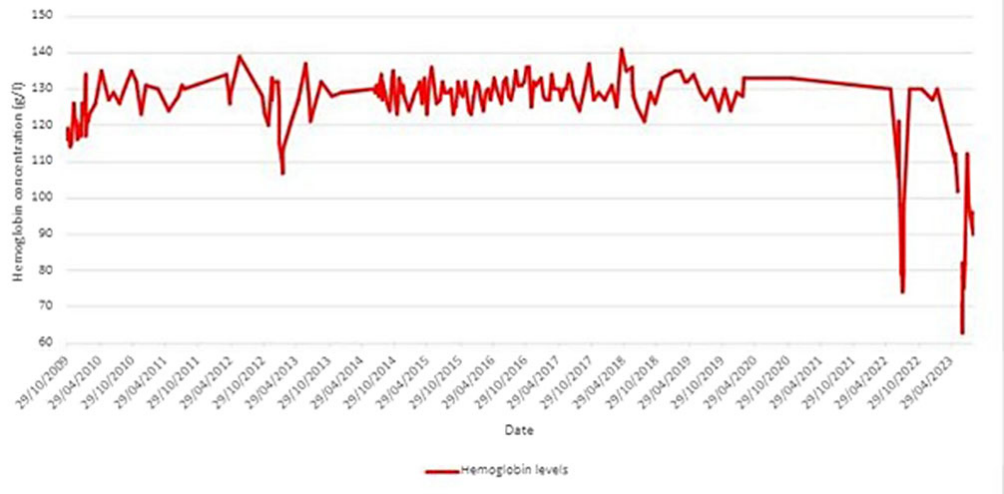

**Conclusions:**

The case was classified as a non-immune haemolytic anaemia secondary to olanzapine and improved with withdrawal of the drug.

**Disclosure of Interest:**

None Declared

